# Noninvasive prediction of T-score in IgA nephropathy using machine learning within the Oxford classification system

**DOI:** 10.3389/fendo.2025.1686670

**Published:** 2026-01-09

**Authors:** Jingyu Dou, Shuhua Jin, Xiaoyue Ma, Lijie Zhang, Lu Wen, Qianqian Li, Jinjin Hai, Bin Yan, Genyang Cheng

**Affiliations:** 1 Department of Nephrology, The First Affiliated Hospital of Zhengzhou University, Zhengzhou, China; 2 Henan Key Laboratory of Imaging and Intelligent Processing, Information Engineering University, Zhengzhou, China

**Keywords:** IgA nephropathy, machine learning, prediction model, radiomics, renal interstitial fibrosis

## Abstract

**Introduction:**

IgA nephropathy (IgAN) is the most common glomerulonephritis worldwide. However, studies utilizing computed tomography (CT) to evaluate the severity of renal interstitial fibrosis in IgAN remain scarce.

**Objective:**

To explore the feasibility and value of combining pretreatment abdominal CT radiomics features with clinical characteristics and machine learning algorithms to determine the Oxford classification T score(renal interstitial fibrosis) of patients with IgAN.

**Methods:**

This retrospective study included 343 patients with IgAN from the First Affiliated Hospital of Zhengzhou University, confirmed by renal biopsy, pretreatment abdominal CT, and clinical data. The patients were divided into training (n = 240) and testing (n = 103) cohorts in a 7:3 ratio. Two senior radiologists delineated the regions of interest, and radiomic features were extracted from the CT images. The extracted radiomic attributes were subjected to least absolute shrinkage and selection operator (LASSO) regression with ten-fold cross-validation, thereby identifying a parsimonious subset of high-weighted imaging biomarkers that confer maximal discriminative power for the prediction of renal interstitial fibrosis. Based on clinical features, radiomic features, or a combination of both, random forest algorithms were employed to construct three-class discrimination models for the Oxford classification T-score of patients with IgAN. The diagnostic performance of the models was evaluated using receiver operating characteristic curves.

**Results:**

After feature selection, 26 radiomics features demonstrated predictive efficacy in diagnosing the T-score and were used to establish the radiomics model. The clinical radiomic model exhibited the best diagnostic performance. To diagnose patients with IgAN of Oxford classification T0, the model achieved an area under the curve (AUC) of 0.94 in the training cohort and 0.94 in the testing cohort. For T1 classification, the AUC was 0.97 in the training and 0.96 in the testing cohorts. For T2 classification, the AUC was 0.94 and 0.95 in the training and testing cohorts, respectively.

**Conclusions:**

The classification diagnostic model based on CT radiomics and clinical features combined with machine learning can accurately predict the Oxford classification T-score in patients with IgAN.

## 1 Introduction

IgA nephropathy (IgAN) is the most common glomerulonephritis worldwide (1). Approximately 40% of patients with IgAN progress to end-stage renal disease (ESRD) within 10–20 years (2). Pathological evaluation is typically required to determine the appropriate treatment strategies to prevent disease progression. In 2009, the International Consensus Oxford Classification categorized IgAN based on histopathological features to predict the prognosis and guide clinical management. This classification was revised in 2017, dividing IgAN into five categories: (1) mesangial hypercellularity (M), (2) endocapillary hypercellularity (E), (3) segmental glomerulosclerosis (S), (4) tubular atrophy/interstitial fibrosis (T), and (5) cellular/fibrocellular crescents (C), each serving as an independent predictor of renal outcomes (3, 4). Renal interstitial fibrosis (T) is characterized by tubular epithelial cell degeneration, atrophy and loss, interstitial lymphocyte and monocyte infiltration, excessive extracellular matrix deposition, and the proliferation of interstitial myofibroblasts (5). It is considered the final common pathway leading to end-stage renal disease (ESRD) and is a crucial biomarker of chronic kidney injury (6). Hernan et al. summarized that M had an independent prognostic value in 5 of 19 studies, E in 4, S in 7, and T in 13 (7). Furthermore, in the constructed prognostic models for IgAN, T lesions demonstrated greater predictive weight than many other clinical and pathological parameters (8). Therefore, the timely assessment of renal fibrosis is crucial for treatment planning and prognosis prediction in patients with IgAN. Renal biopsy remains the gold standard for the diagnosis of renal fibrosis (9). However, it is associated with certain risks, including the formation of arteriovenous fistulas, massive hematuria, perirenal hematomas, and in severe cases, it may even lead to death (10). Additionally, some patients refuse to undergo biopsies due to fear. Moreover, the histopathological diagnosis only reflects the disease status at the time of biopsy and is unsuitable for longitudinal follow-up.

Radiomics enables the extraction and quantification of high-throughput imaging biomarkers that extend beyond human perceptual capabilities. By integrating these biomarkers with various machine learning (ML) techniques, radiomics can effectively identify subtle and complex tissue alterations (11). In recent times, radiomics research has witnessed remarkable expansion. It comprehensively integrates knowledge from multiple disciplines, namely radiology, computer science, and data analysis, with the aim of extracting quantitative features from medical images. This interdisciplinary approach has enabled a more in - depth exploration of the information contained within medical images, facilitating a more accurate understanding of diseases and potentially revolutionizing the field of medical diagnosis and treatment (12). This approach has been successfully applied in the identification and differentiation of renal diseases, including: discrimination of renal tumors (13) and their histological subtypes (14), detection of early renal injury in patients with diabetic (15), differential diagnosis of chronic kidney disease (CKD) (16), among others.

Radiomics-based machine learning classifiers have demonstrated potential value for the noninvasive diagnosis of IgAN with or without crescents (17) and have shown high accuracy in assessing lupus nephritis activity (18), confirming the utility of radiomics in renal pathology evaluation. Compared to ultrasound, computed tomography (CT) provides higher spatial resolution images with superior anatomical detail and is less affected by gas interference. To the present, a substantial body of research has corroborated the diagnostic and predictive efficacy of CT texture analysis across a diverse range of pathologies. This encompasses aspects such as the differentiation of tumors, the grading of lesions, and the pathological characterization of various conditions. These investigations have not only provided in - depth insights into the potential of CT texture analysis in understanding disease states but have also laid a solid foundation for its further application and refinement in clinical practice and research (19–21). However, studies utilizing CT to evaluate the severity of renal interstitial fibrosis (T score) in IgAN remain scarce.

Thus, the present study aimed to develop and validate a machine learning algorithm combining abdominal CT radiomics and clinical parameters for the precise evaluation of the Oxford T classification in IgA nephropathy.

## 2 Methods

### 2.1 Study design and population

**Figure 1:**
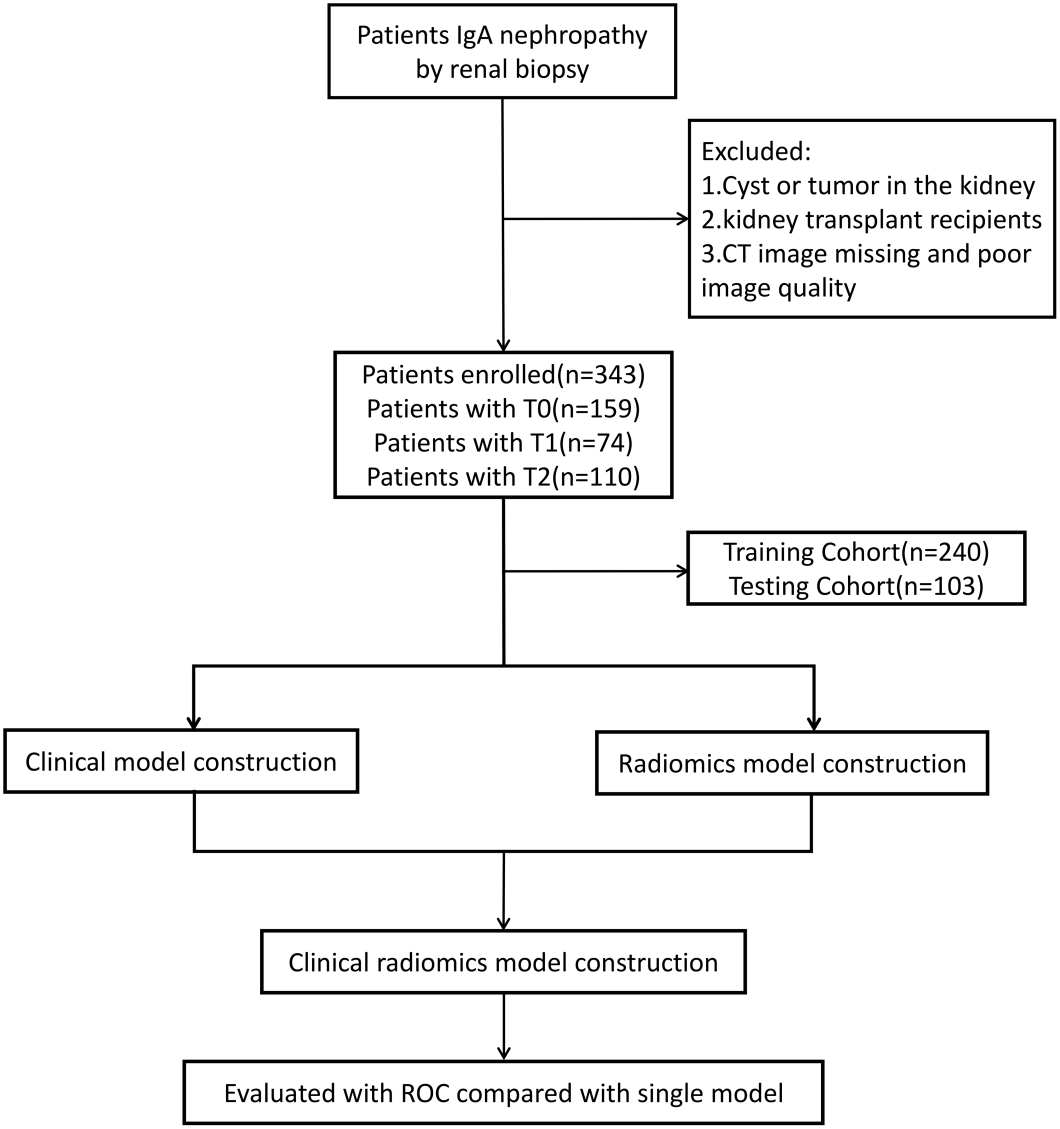
The flow diagram of the study.

A total of 343 patients diagnosed by renal biopsy at the First Affiliated Hospital of Zhengzhou University between January 2019 and June 2023 were retrospectively enrolled. The inclusion criteria were as follows: (1) IgAN confirmed by renal puncture biopsy. 2) Abdominal CT scan performed within 3 days prior to renal biopsy. 3) No prior treatment with corticosteroids, immunosuppressants, antihypertensive agents, or lipid-lowering agents. The exclusion criteria were as follows: 1) Patients with renal calculi, cysts, hydronephrosis, or renal tumors.2) Kidney transplant recipients. 3)Poor-quality imaging data. The following baseline data were collected: age, sex, blood pressure, systolic blood pressure (SBP), diastolic blood pressure (DBP), serum uric acid, creatinine, estimated glomerular filtration rate (eGFR), hemoglobin (HB), blood urea nitrogen, total protein, serum albumin,24-hour urinary protein excretion, total cholesterol, and lipid profile. Patients were randomly divided into training (n = 240, 70%) and validation cohorts (n = 103, 30%) based on the Oxford Classification (T0, T1, T2) derived from renal biopsy results. This study complied with the Declaration of Helsinki and was approved by the Ethics Committee of First Affiliated Hospital of Zhengzhou University. A flowchart of the study is shown in **Figure 1**.

### 2.2 Abdominal CT examination

All patients underwent abdominal CT scanning using a GE Discovery CT750 HD scanner with the following parameters: Tube voltage: 120 kV; Tube current: Automatic modulation; Pitch: 0.75–1.50; Collimation: 64 × 0.625 mm; Field of view (FOV): 350 × 350 mm; Matrix size: 512 × 512; Slice thickness: 5 mm (acquisition), 1 mm (reconstruction). All scans were performed by board-certified radiologists with ≥10 years of experience in abdominal imaging.

### 2.3 Renal biopsy

Renal biopsy was performed within 3 days of renal CT examination by two experienced pathologists. The right kidney was selected for a biopsy. Paraffin-embedded sections were stained with hematoxylin and eosin, periodate Schiff’s solution, trimethylamine silver, and Masson’s trichrome. Biopsy specimens from all patients were evaluated by immunofluorescence, light, or electron microscopy. The pathological variables of IgAN were scored according to the MEST-C criteria: mesangial cell increase, capillary cell increase, segmental glomerulosclerosis, tubular atrophy/interstitial fibrosis, and the presence of crescents. Tubular atrophy/interstitial fibrosis (T) is graded as follows: T0: none, or involvement of <25% of the cortical area; T1: involvement of 25%–50% of the cortical area; and T2: involvement of >50% of the cortical area. Any discrepancies between the two pathologists were resolved by consensus through panel discussion.

### 2.4 CT image segmentation and feature extraction

The acquired CT images were imported in DICOM format into ITK-SNAP for manual image segmentation. This segmentation was performed independently by two senior radiologists. Any discrepancies between the interpretations of the two raters were resolved through a consensus discussion within the panel. We evaluated the selected region of interest (ROI) within the renal cortex, followed by image processing. The CT images were processed using built-in filters, such as wavelet transform and Laplacian of Gaussian (LoG) filters, to generate derived images. The core steps include: (1) Isotropic resampling: following the IBSI standardization guidelines (22), we resampled all image voxels to an isotropic 1×1×1 mm³ spacing using B-spline interpolation. (2) Wavelet Transform: We employed the Coiflets wavelet. Compared to ‘haar’ or ‘db’ wavelets, the Coiflets wavelet offers better symmetry and regularity, which helps reduce artifacts during decomposition and reconstruction (23). We performed a 3-level discrete wavelet decomposition. This generated 8 sets of filtered images: LLL, LLH, LHL, LHH, HLL, HLH, HHL, and HHH. Radiomic features were subsequently extracted from all 8 sub-band images independently. (3) LoG Filter: We applied a discretized 2D/3D Laplacian of Gaussian operator. The LoG filter is an edge-enhancement and blob-detection filter. It highlights edges and blob-like structures at different scales by applying Gaussian smoothing followed by the Laplacian operator, making it highly sensitive to texture coarseness (24). We used four different sigma (σ) values: 1.0, 2.0, 3.0, and 5.0 mm. The original image was filtered separately with LoG kernels using these four σ values, resulting in four sets of filtered images, from which features were extracted independently. (4) Gray-level Intensity Discretization: In accordance with the IBSI guideline (22), image intensities were discretized using a fixed bin width corresponding to 32 gray levels. Radiomic features were subsequently extracted from both the original and the derived images for each patient (25). A preprocessing pipeline was applied to the extracted features. Features with poor intra-observer or inter-observer reproducibility (i.e., intraclass correlation coefficient [ICC] < 0.75) were excluded, ensuring that only robust features were retained for subsequent analysis. Finally, the least absolute shrinkage and selection operator (LASSO) algorithm was applied to select the most predictive features for the construction of the subsequent model.

### 2.5 Construction of predictive models

We developed three distinct machine learning models to predict Oxford Classification T scores in IgA nephropathy: (1) clinical model: utilizing only clinical parameters (2) radiomics model: based exclusively on imaging features (3) integrated clinical radiomics model combining clinical and radiomic features. All models were constructed using the Random Forest (RF) algorithm, which was chosen for its robustness in handling high-dimensional data and its inherent feature importance evaluation capabilities. The predictive performance of each model was systematically evaluated and compared using the receiver operating characteristic (ROC) curve analysis.

### 2.6 Statistical analysis

Radiomic features were extracted using PyRadiomics version 3.0.1. All subsequent data processing and model construction were performed in Python 3.7.4 utilizing libraries including Scikit-learn 1.0.2, NumPy 1.21.6, SciPy 1.7.3, Pandas 1.3.5, and Matplotlib 3.5.3. Statistical analyses were conducted using IBM SPSS Statistics (Version 26.0). All tests were two-sided, and a P-value < 0.05 was considered statistically significant. Normally distributed continuous data are presented as mean ± standard deviation, while non-normally distributed data are expressed as median (25th - 75th percentiles). Categorical variables were compared using the Chi-square test. For continuous variables, normality and homogeneity of variances were assessed using the Kolmogorov-Smirnov test and Levene’s test, respectively. Based on the results of these tests, either one-way analysis of variance (ANOVA) or the Kruskal-Wallis H test was used for comparisons.

## 3 Results

### 3.1 Baseline characteristics of the participants

This study enrolled 343 patients with IgA nephropathy and stratified them into three groups based on the Oxford Classification T scores: T0 (n=159), T1 (n=74), and T2 (n=110). The cohort comprised 204 males and 139 females with a mean age of 38 years. The patients were randomly allocated to either the training (n=240) or the testing (n=103) cohorts.

As presented in **Tables 1**, **2**, the clinical characteristics were significantly different (P<0.05) among the T-score groups for the following parameters: eGFR, blood urea nitrogen (UREA), serum creatinine (Scr), uric acid (UA), SBP, DBP, HB, and 24-hour total proteinuria (24hTP). No statistically significant differences were observed in the remaining clinical indicators.

**Table 1:** Comparison of baseline clinical characteristics among three groups in the training cohort.

Clinical factors	T0 (n=106)	T1 (n=50)	T2 (n=84)	X^2^/F/H	*P-value*
Age(years)	35 (27-46)	35 (27-46)	32 (26-40)	4.28	0.12
Sex				2.788^b^	0.248
Male	60	27	56		
Female	46	23	28		
Hemoglobin(g/L)	127.4	126	118	27.648	<0.01
	(118.0-141.5)	(109.8-143)	(105-131)		
Systolic pressure (mmHg)	135.01 ± 20.804	137.63 ± 18.620	146.60 ± 18.990	39.536^a^	<0.01
Diastolic pressure (mmHg)	89.38 ± 14.008	90.18 ± 13.259	94.93 ± 14.727	7.741^a^	<0.01
Uric acid(mmol/L)	341.80 ± 103.262	341.80 ± 103.262	341.80 ± 103.262	29.59^a^	<0.01
Total protein(g/L)	62.95	61.30	59.65	5.15	0.076
	(55.00-67.03)	(56.78-65.63)	(55-64.95)		
Albumin(g/L)	38.1	37.8	36.4	5.16	0.076
	(34.33-41.05)	(34.3-41.2)	(33.68-39.55)		
Totalcholesterol(mmol/L)	4.63	4.7	4.8	0.385	0.83
	(3.93-5.72)	(4.08-6.02)	(3.97-5.55)		
Triglycerides(mmol/L)	1.53	1.61	1.65	3.33	0.19
	(1.02-2.32)	(1.03-2.37)	(1.20-2.46)		
Urea(mmol/L)	6.08	8.33	11.73	157.33	<0.01
	(4.70-7.82)	(6.1-11.4)	(9.20-13.99)		
Creatinine level(mmol/L)	86	113.50	207.00	192.42	<0.01
	(67.5-109.5)	(83.25-162.25)	(143.50-268.75)		
eGFR(mL/min/1.73m^2^)	94.76	57.35	31.50	204.38	<0.01
	(62.95-117.77)	(40.41-84.92)	(24.25-47.82)		
24h Urine protein(g/24H)	1.61	2.05	3.00	46.49	<0.01
	(0.84-2.68)	(1.07-3.78)	(1.68-4.58)		

^a^represents F-value, ^b^represents χ²-value, and the remaining test statistics are H-values.

**Table 2:** Comparison of baseline clinical characteristics among three groups in the testing cohort.

Clinical factors	T0 (n=53)	T1 (n=24)	T2 (n=26)	X^2^/F/H	*P-value*
Age(years)	34 (27-46)	35 (27-45)	33 (27-40)	4.07	0.131
Sex				3.99^b^	0.136
Male	30	13	18		
Female	23	11	8		
Hemoglobin(g/L)	128	126	121	14.915	<0.01
	(118-143)	(110-144)	(108-133)		
Systolic pressure (mmHg)	135.43 ± 21.56	137.01 ± 17.95	146.24 ± 17.86	29.59^a^	<0.01
Diastolic pressure (mmHg)	89.38 ± 14.21	90.07 ± 13.11	95.33 ± 13.92	12.36^a^	0.002
Uric acid(mmol/L)	347.08 ± 103.84	398.37 ± 123.45	441.86 ± 103.53	49.63^a^	<0.01
Total protein(g/L)	62.90	61.30	60.85	2.12	0.347
	(55.00-67.60)	(56.95-65.80)	(58.10-66.80)		
Albumin(g/L)	38.10	37.50	37.85	1.58	0.455
	(34.50-41.10)	(33.95-41.35)	(35.10-40.63)		
Totalcholesterol(mmol/L)	4.57	4.69	4.79	0.51	0.775
	(3.90-5.72)	(4.09-5.93)	(4.03-5.40)		
Triglycerides(mmol/L)	1.56	1.60	1.57	1.95	0.377
	(1.02-2.29)	(0.98-2.38)	(1.20-2.53)		
Urea(mmol/L)	6.10	8.64	10.60	97.96	<0.01
	(4.91-7.86)	(6.33-11.40)	(8.80-13.62)		
Creatinine level(mmol/L)	88.00	123.00	204.00	126.74	<0.01
	(68.00-111.50)	(92.00-163.50)	(135.30-261.50)		
eGFR(mL/min/1.73m^2^)	92.87	54.84	31.84	136.72	<0.01
	(64.88-117.39)	(40.09-87.24)	(25.26-53.11)		
24h Urine protein(g/24H)	1.61	1.85	2.40	27.42	<0.01
	(0.85-2.64)	(1.02-3.51)	(1.26-4.18)		

^a^represents F-value, ^b^represents χ²-value, and the remaining test statistics are H-values.

### 3.2 Feature selection and model development

Following inter- and intra-observer consistency testing, 652 of 1504 features were excluded owing to intraclass correlation coefficient (ICC) values < 0.75, leaving 852 robust features for subsequent analysis. In the radiomics model, the remaining 852 radiomic features after the aforementioned procedures were first subjected to LASSO regression for dimensionality reduction and then fed into a random forest algorithm for model construction. In the clinical model, 14 clinical variables were directly entered into the random forest algorithm without prior feature selection. In the combined model, the 852 retained radiomic features and the 14 clinical variables were jointly subjected to LASSO regression screening, followed by random forest modeling. **Table 3** illustrates the final set of features incorporated into the clinic-radiomic model. **Table 4** presents the included radiomics features with a brief explanation.

**Table 3:** Extracted features by LASSO regression in clinical-radiomics model.

Model	Feature name
RF	wavelet-LHL_glcm_ClusterShade wavelet-LHL_glcm_Correlation wavelet-LHH_gldm_DependenceNonUniformityNormalized wavelet-HLH_gldm_DependenceNonUniformityNormalized wavelet-HHH_glcm_Correlation original_gldm_DependenceEntropy log-sigma-5-0-mm-3D_firstorder_90Percentile log-sigma-5-0-mm-3D_firstorder_Uniformity log-sigma-5-0-mm-3D_glcm_InverseVariance log-sigma-5-0-mm-3D_glcm_JointEnergy log-sigma-5-0-mm-3D_gldm_DependenceEntropy log-sigma-5-0-mm-3D_glrlm_ShortRunEmphasis log-sigma-5-0-mm-3D_glszm_ZoneEntropy wavelet-LHL_gldm_LargeDependenceLowGrayLevelEmphasis wavelet-LHL_glrlm_ShortRunEmphasis wavelet-LHH_glrlm_GrayLevelNonUniformityNormalized wavelet-LHH_glrlm_ShortRunEmphasis wavelet-HLL_gldm_LargeDependenceEmphasis wavelet-HLL_glszm_SmallAreaEmphasis wavelet-HLL_ngtdm_Busyness wavelet-HLH_gldm_DependenceVariance wavelet-HLH_ngtdm_Busyness wavelet-HHL_glcm_MCC wavelet-HHL_glszm_GrayLevelNonUniformityNormalized wavelet-HHH_glszm_GrayLevelNonUniformityNormalized wavelet-LLH_glcm_ClusterProminence Urinary ptotein quantitative eGFR

**Table 4:** Explanation of radiomic features.

Feature name	Brief explanation	Potential link to macroscopic CT findings	Potential clinical significance in assessing renal interstitial fibrosis
wavelet-LHL_glcm_ClusterShade	Cluster Shade: Measures the asymmetry of the gray-level distribution. A higher value indicates greater asymmetry (skewness).	Reflects the “skewness” of pixel intensity distribution in a local region.	Fibrosis disrupts normal architecture, potentially leading to irregular and asymmetric CT value distributions, which this feature may capture.
wavelet-LHL_glcm_Correlation	Correlation: Measures the linear dependency of gray levels of neighboring pixels. High values indicate directional, homogeneous textures.	Reflects the regularity and directional consistency of local tissue structure.	RIF (e.g., streaky, patchy changes) may disrupt the normal, uniform kidney texture, reducing local correlation.
wavelet-LHH_gldm_ DependenceNonUniformityNormalized	Dependence Non-Uniformity Normalized: Measures the uniformity of the dependence distribution. Lower values indicate a more homogeneous distribution.	Reflects the spatial homogeneity of “coarse” or “fine” texture patterns.	Fibrosis may introduce heterogeneous, uneven coarse texture areas (e.g., interlacing fibrous bands and residual tissue), altering this feature.
wavelet-HLH_gldm_ DependenceNonUniformityNormalized	(Same as above, but in a different filter direction)	Captures variations in texture uniformity at different scales/orientations.	This feature helps comprehensively assess the multi-directional structural disruption caused by fibrosis.
Feature Name	Brief Explanation	Potential Link to Macroscopic CT Findings	Potential Clinical Significance in Assessing Renal Interstitial Fibrosis
wavelet-HHH_glcm_Correlation	Correlation​ (in the high-frequency​ sub-band). High frequency represents detail and edge information.	represents detail and edge information.Reflects the directional consistency of fine structures (e.g., small interfaces, micro-nodules).	Advanced fibrosis or associated inflammation/edema may create anisotropic changes at a micro-scale, sensitively detected by this feature.
original_gldm_DependenceEntropy	Measures the randomness/complexity of dependence patterns. Higher entropy indicates more complex, unpredictable patterns.	Reflects the complexity and disorder of tissue texture.	Fibrosis is a complex process that may increase structural complexity (e.g., admixture of normal tissue, fibrosis, atrophy), potentially increasing entropy.
log-sigma-5-0-mm-3D_firstorder_90Percentile	90th Percentile: The intensity value at the 90th percentile of the filtered image histogram. Reflects high-intensity regions.	Captures the intensity level of relatively hyperdense​ areas.	May relate to slight density increases from early collagen deposition/inflammatory infiltration, or the relative prominence of vascular structures.
log-sigma-5-0-mm-3D_firstorder_Uniformity	Uniformity: Measures the uniformity of the intensity value distribution. Higher values indicate more uniform intensities.	Reflects the overall homogeneity of the image after LoG filtering (highlighting structures at a 5mm scale).	Fibrosis-induced heterogeneity may decrease the uniformity of the 5mm-scale filtered image.
Feature Name	Brief Explanation	Potential Link to Macroscopic CT Findings	Potential Clinical Significance in Assessing Renal Interstitial Fibrosis
log-sigma-5-0-mm-3D_glcm_InverseVariance	Inverse Variance: Less sensitive to local gray-level differences. Emphasizes homogeneous areas, suppresses high-contrast areas.	Sensitive to large, uniform regions.	The homogeneity of renal cortex/medulla is disrupted by fibrosis, potentially decreasing this feature. Complements Uniformity.
log-sigma-5-0-mm-3D_glcm_JointEnergy	JMeasures the uniformity of the gray-level distribution. Higher values indicate simpler, more homogeneous textures.	Reflects the “regularity” and “predictability” of texture.	Normal kidney has relatively regular texture. Fibrosis introduces irregularity, potentially decreasing energy.
log-sigma-5-0-mm-3D_gldm_DependenceEntropy	Dependence Entropy​ (at the 5mm scale).	Assesses texture complexity/disorder at a specific medium scale (5mm).	May help localize medium-scale structural disorder associated with fibrotic plaques of millimeter size.
log-sigma-5-0-mm-3D_glrlm_ShortRunEmphasis	Short Run Emphasis: Emphasizes the prevalence of short runs​ (short sequences of consecutive same gray levels). High values indicate fine, granular textures.	Reflects the abundance of fine, granular​ textures.	On non-contrast CT, fibrosis may present as subtle granular or ground-glass opacities, potentially increasing this feature.
Feature Name	Brief Explanation	Potential Link to Macroscopic CT Findings	Potential Clinical Significance in Assessing Renal Interstitial Fibrosis
log-sigma-5-0-mm-3D_glszm_ZoneEntropy	Zone Size Entropy: Measures the randomness in the distribution of zone sizes (homogeneous areas). High entropy indicates heterogeneous, complex zone sizes.	Reflects the heterogeneity of homogeneous area​ sizes.	Fibrosis partitions tissue into irregular functional areas of varying size, increasing complexity in zone size distribution, raising entropy.
wavelet-LHL_gldm_ LargeDependenceLowGrayLevelEmphasis	​ Large Dependence Low Gray Level Emphasis: Emphasizes large, low-intensity​ areas.	Sensitive to large, hypodense​ regions.	May correlate with larger areas of low density formed in late-stage fibrosis with tubular atrophy and expanded interstitium.
wavelet-LHL_glrlm_ShortRunEmphasis	Short Run Emphasis​ (in the LHL​ sub-band).	Captures fine texture at a specific frequency/orientation combination.	Assesses microscopic textural changes from fibrosis from multiple angles (frequency + orientation).
wavelet-LHH_glrlm_ GrayLevelNonUniformityNormalized	Gray Level Non-Uniformity Normalized: Measures the similarity of run lengths across gray levels. Lower values indicate greater uniformity.	Reflects the uniformity of texture across different gray levels.	Fibrosis causes uneven texture distribution across areas of different CT values (e.g., water, soft tissue), increasing non-uniformity.
Feature Name	Brief Explanation	Potential Link to Macroscopic CT Findings	Potential Clinical Significance in Assessing Renal Interstitial Fibrosis
wavelet-LHH_glrlm_ShortRunEmphasis	This feature quantifies the proportion of short gray-level runs in the wavelet-LHH layer, reflecting fine, fragmented textures.	Higher values correspond to discontinuous, short linear or dot-like patterns on non-contrast CT.	An elevated value indicates early micro-structural disruption in renal interstitial fibrosis, enabling non-invasive detection of early fibrotic change.
wavelet-HLL_gldm_ LargeDependenceEmphasis	Large Dependence Emphasis: Emphasizes areas with large​ gray-level dependencies, regardless of intensity.	Sensitive to large, homogeneous regions​ in the image.	May reflect relatively homogeneous fibrotic scar regions formed during disease progression.
wavelet-HLL_glszm_SmallAreaEmphasis	Small Area Emphasis: Emphasizes small-sized​ homogeneous zones.	Reflects the abundance of tiny, scattered​ structures.	May relate to early, scattered punctate fibrotic foci or residual small normal tissue areas.
wavelet-HLH_gldm_DependenceVariance	Dependence Variance: The variance in dependence values. High variance indicates a wide range of coarseness/fineness.	Reflects the magnitude of variation in texture “coarseness” patterns.	Fibrotic areas may be internally heterogeneous, containing a mix of fine to coarse patterns, increasing variance.
Feature Name	Brief Explanation	Potential Link to Macroscopic CT Findings	Potential Clinical Significance in Assessing Renal Interstitial Fibrosis
wavelet-HLH_ngtdm_Busyness	This feature quantifies the “busy” rate of gray-level differences between pixels and their neighborhood in the wavelet-HLH layer.	Higher values yield speckled or jagged, rapidly fluctuating textures on non-contrast CT.	An elevated value signals active interstitial fibrosis with prominent inflammation, allowing non-invasive monitoring of disease activity.
wavelet-HHL_glcm_MCC	Maximal Correlation Coefficient: Measures the strength of linear structures potentially present in the image.	Sensitive to potential directional, linear structures​ in the image.	In the kidney, may capture altered linear alignment of tubules/vessels due to fibrotic traction, or the fibrous bands themselves.
wavelet-HHL_glszm_ GrayLevelNonUniformityNormalized	This feature quantifies the non-uniformity in the size distribution of graylevel zones within a wavelet-transformed image sub-band, reflecting the heterogeneity of texture.	Measures the similarity of gray-level intensities within homogeneous zones. Lower values indicate uniform intensities within zones.	Fibrosis may cause gray-level variations within a single “homogeneous zone” (e.g., mix of collagen, cells, edema), decreasing this value.
Feature Name	Brief Explanation	Potential Link to Macroscopic CT Findings	Potential Clinical Significance in Assessing Renal Interstitial Fibrosis
wavelet-HHH_glszm_ GrayLevelNonUniformityNormalized	This feature quantifies the unevenness of gray-level–zone distribution in the wavelet-HHH layer.	This feature quantifies microscopic texture heterogeneity, which serves as an imaging biomarker for the macroscopic complexity and non-uniformity visually observed in CT findings.	This feature’s measured heterogeneity is a quantitative imaging biomarker that can non-invasively assess the severity and spatial complexity of renal interstitial fibrosis, aiding in disease staging and monitoring progression.
wavelet-HLL_ngtdm_Busyness	This feature quantifies the rapid local gray-level variations and coarseness in an image after high-frequency wavelet filtering.	An increase in its value may correspond to coarse, heterogeneous texture patterns observed in the renal cortex on CT images.	It could serve as an imaging biomarker for quantifying renal interstitial fibrosis, with higher values suggesting greater disruption of tissue microarchitecture associated with fibrosis severity.
wavelet-LLH_glcm_ClusterProminence	Cluster Prominence: Similar to Cluster Shade, measures asymmetry and peakedness of the distribution. High values indicate a skewed, peaked distribution.	May correspond to the CT value distribution characteristic of a dominant tissue type (e.g., a large fibrotic area).	Elevated values of this LLH wavelet GLCM feature indicate textural asymmetry and clustering, which can quantify the heterogeneous distribution and severity of fibrotic tissue in renal interstitial fibrosis.

### 3.3 Performance of the clinical diagnostic model

**Figure 2:**
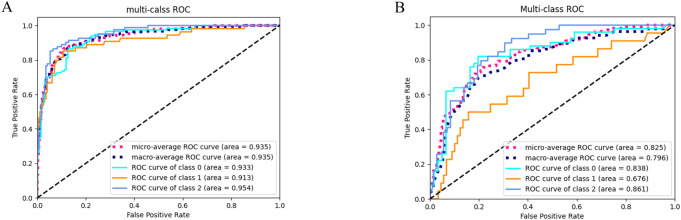
ROC curves of the clinical model for three-category T-stage classification. **(A)** ROC curves of the classification model in the training cohort. **(B)** ROC curves of the classification model in the testing cohort.

**Figure 2** displays the ROC curves of the random forest clinical diagnostic model for Oxford T-score stratification in IgAN, demonstrating performance with training cohort AUCs of 0.933 (T0), 0.913 (T1), and 0.954 (T2), supported by both micro-average and macro-average AUCs of 0.935, while testing cohort results showed AUCs of 0.838 (T0), 0.676 (T1), and 0.861 (T2) with corresponding micro-average (0.825) and macro-average (0.796) metrics.

### 3.4 Performance of the radiomics diagnostic model

**Figure 3:**
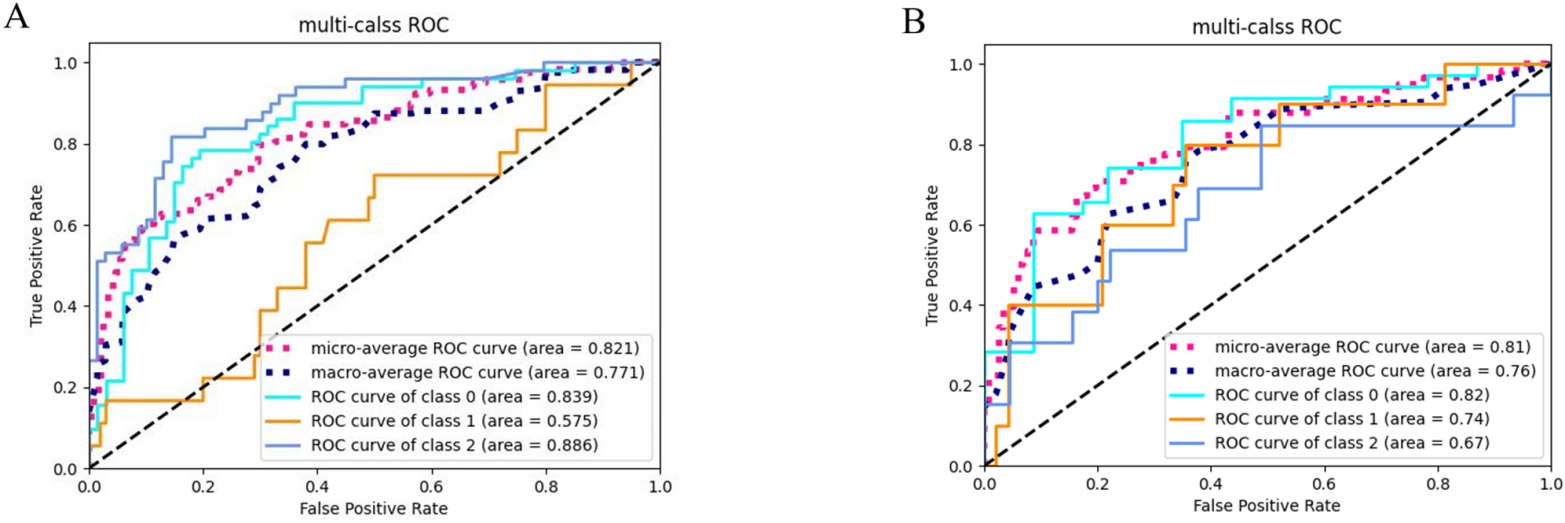
ROC curves of the radiomics model for three-category T-stage classification. **(A)** ROC curves of the classification model in the training cohort. **(B)** ROC curves of the classification model in the testing cohort.

**Figure 3** presents the receiver operating characteristic (ROC) curves of the random forest-based radiomics model for Oxford classification T-score stratification in patients with IgA nephropathy, demonstrating robust performance with area under the curve (AUC) values of 0.84 (T0), 0.58 (T1), and 0.89 (T2) in the training cohort (micro-average AUC: 0.82; macro-average AUC: 0.77), while maintaining diagnostic capability in the testing cohort with AUCs of 0.82 (T0), 0.74 (T1), and 0.67 (T2) (micro-average AUC: 0.81; macro-average AUC: 0.76).

### 3.5 Performance of the clinical-radiomics diagnostic model

**Figure 4:**
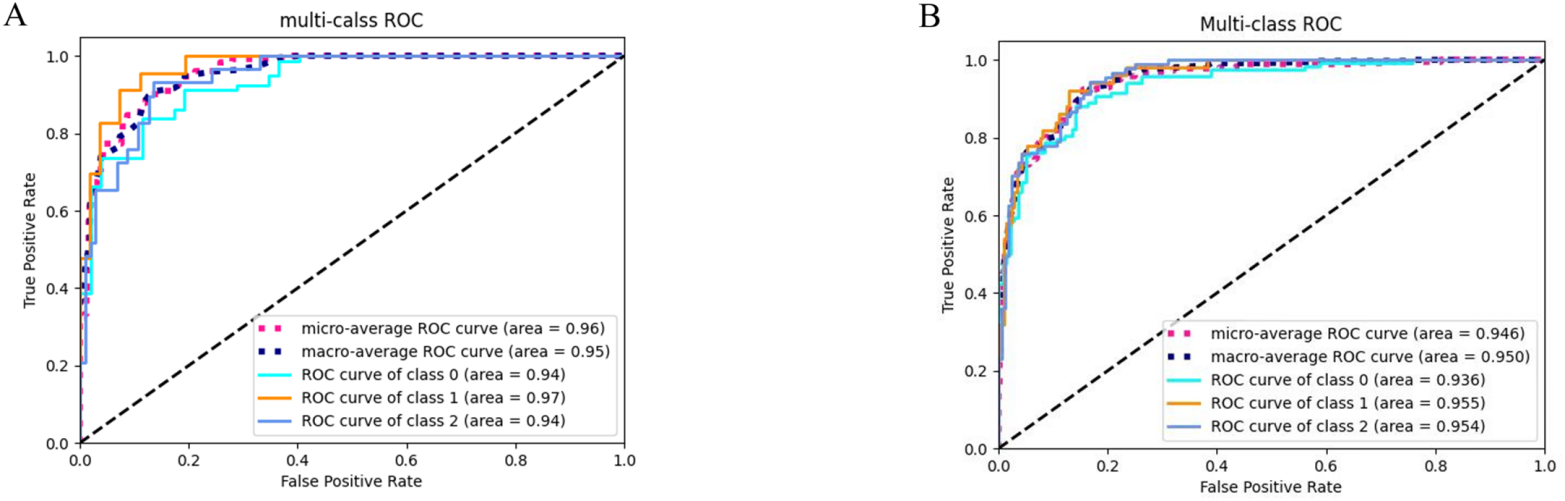
ROC curves of the clinical-radiomics model for three-category T-stage classification. **(A)** ROC curves of the classification model in the training cohort. **(B)** ROC curves of the classification model in the testing cohort.

**Figure 5:**
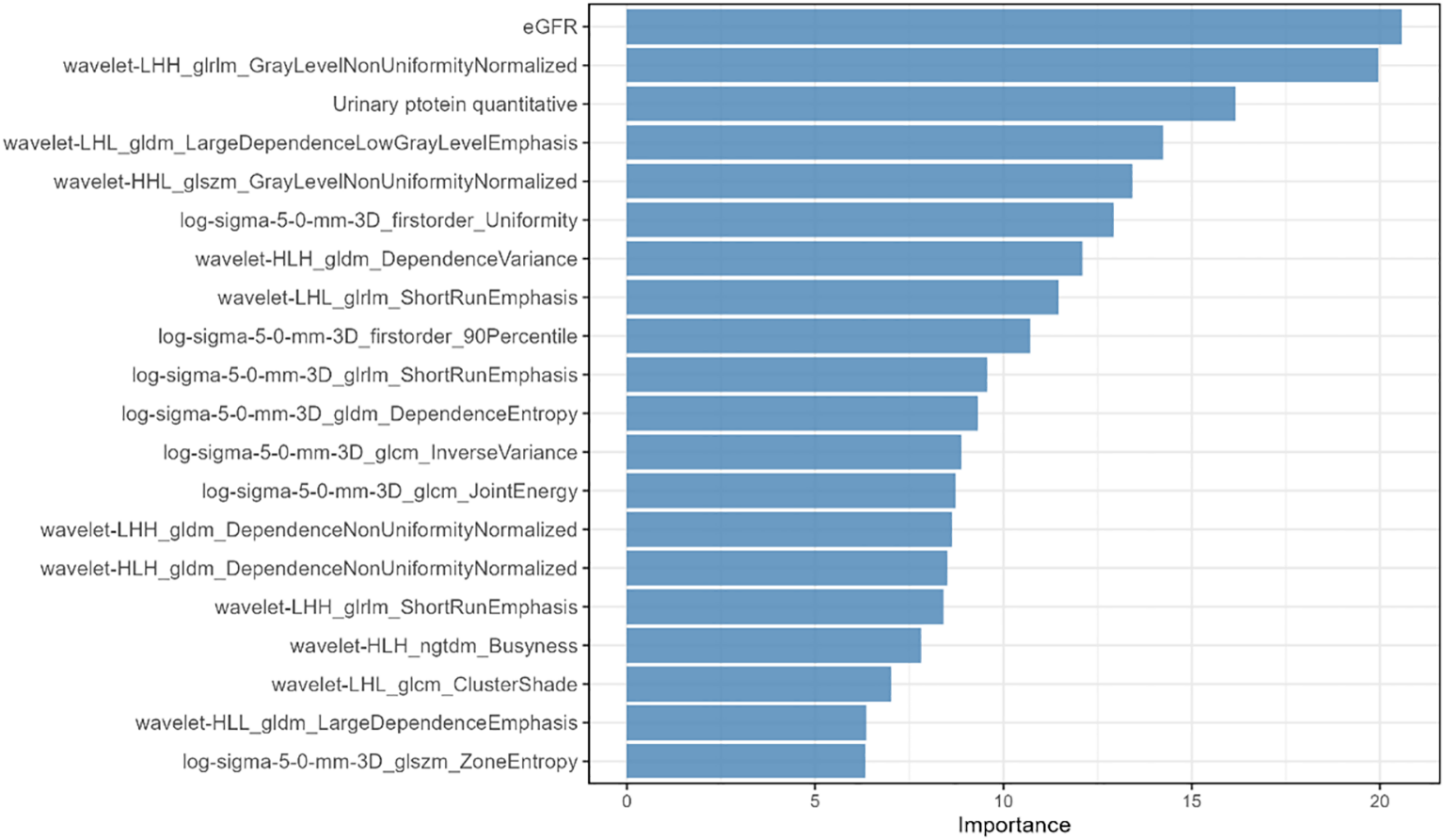
Top-twenty feature importance of the three-class clinical-radiomic diagnostic model.

**Figure 4** demonstrates the receiver operating characteristic (ROC) curves of the integrated clinical-radiomics random forest model for Oxford classification T-score stratification in IgA nephropathy, achieving exceptional discriminative performance with AUC values of 0.94 (T0), 0.97 (T1), and 0.94 (T2) in the training cohort (micro-average AUC: 0.96; macro-average AUC: 0.95), with maintained high accuracy in the testing cohort (AUCs: 0.94 [T0], 0.96 [T1], 0.95 [T2]; micro-average: 0.95; macro-average: 0.95). **Figure 5** illustrates​ the top 20 features in importance for the clinical-radiomic model, ranked by importance.

## 4 Discussion

Recent advances in radiomics and the growing trend of multidisciplinary integration have created significant opportunities and challenges for the application of AI in medical imaging. Artificial intelligence demonstrates superior diagnostic performance compared to conventional radiological assessment while simultaneously enhancing radiologists’ diagnostic accuracy and efficiency when used as an assistive tool. Jiang et al. (26) developed a support vector machine model utilizing multiparametric MRI to evaluate chronic kidney disease and renal interstitial fibrosis, achieving an AUC of 0.91 for assessing fibrosis severity. Bandara et al. (27) established an ultrasound-based radiomics model for CKD detection with promising performance. Collectively, these studies demonstrated the strong potential of various AI algorithms in renal radiomics applications. However, studies have indicated that the accuracy of ultrasonography is operator-dependent, and abnormal echogenicity often only becomes apparent in cases of severe dysfunction (16, 28). Meanwhile, MRI is associated with higher costs and has limitations in certain patient populations, such as those with metallic implants or claustrophobia (29). Computed tomography (CT) can serve as an imaging modality within a multimodal approach for assessing renal fibrosis. Cohen EP et al. applied CT-based radiomics to analyze the renal cortex in non-human primates that had undergone radiotherapy, demonstrating its role in quantifying renal fibrosis. Nevertheless, there is limited research on using CT imaging to evaluate renal interstitial fibrosis (IF) specifically in patients with IgA nephropathy (IgAN). Therefore, this study focuses on the potential of CT-based radiomics for this purpose. By integrating radiomics with key clinical lab indices (eGFR, 24-h proteinuria), we built a clinic-radiomic model to predict the Oxford T score (renal interstitial fibrosis) in IgA nephropathy.

However, while the existing research has predominantly focused on binary classification (distinguishing between two disease states or the presence or absence of a pathology), few studies have addressed multiclass discrimination. Building on this foundation and with a focus on enhanced clinical relevance, this study employed a random forest algorithm to perform a three-category classification of the Oxford Classification T scores in IgA nephropathy. The Random Forest (RF) algorithm has been widely adopted in the development of models for CKD identification and prognosis prediction (30–32) because of its robust classification and regression capabilities in handling complex datasets. This ensemble learning method excels in capturing nonlinear relationships among multiple variables while enhancing prediction interpretability through feature importance analysis. Notably, comparative studies have consistently demonstrated superior accuracy of RF over alternative machine learning algorithms (31, 33, 34), establishing it as the method of choice in medical prediction tasks. Therefore, this study aimed to develop and validate a clinical-radiomics model based on the random forest algorithm to predict the T-scores of the Oxford Classification (representing renal interstitial fibrosis) in patients with IgA nephropathy (IgAN). In this study, we constructed three distinct models using a random forest algorithm: one based solely on radiomic features, another on clinical features alone, and a combined model integrating both. The clinical model and the combined clinico-radiomic model demonstrated comparable performance in the training set, with AUC values ranging from 0.91 to 0.97, indicating excellent predictive ability. Conversely, the radiomics model showed unsatisfactory performance for classifying T1 lesions (AUC = 0.58) in the training set. In the testing set, the combined clinico-radiomic model exhibited superior performance for classifying all categories of the Oxford Classification in IgA nephropathy patients, achieving AUC values between 0.94 and 0.96, which was higher than those of the clinical and radiomics models alone. Specifically, the clinical model demonstrated a limited ability to classify T1 lesions in the testing set (AUC = 0.68), while the radiomics model performed poorly in classifying T2 lesions (AUC = 0.67). The optimal performance of the combined model highlights the complementary value of both feature types: radiomics provides objective, quantifiable, and high-dimensional data (albeit with limited interpretability), and the radiomic feature set selected in this study collectively constructs a comprehensive model capable of quantifying the heterogeneity of non-contrast kidney CT images from multiple dimensions. These features (e.g., various entropy, non-uniformity, short-run emphasis, and cluster features) jointly reflect the increased organizational complexity, disorder, and multi-scale textural changes induced by renal interstitial fibrosis. Specifically, wavelet-based features enable multi-directional, multi-frequency detection of subtle ground-glass opacities, medium-sized fibrotic plaques, and large-scale structural distortions, while features at specific spatial scales (e.g., post-LoG filtered features) capture pathological changes corresponding to those scales. They not only have potential quantitative correlations with traditional macroscopic CT findings (such as irregular contours, cortical coarseness, and density inhomogeneity) but, more importantly, can more sensitively reveal microstructural disorganization before morphological changes become apparent. Therefore, this feature set holds promise as a non-invasive, quantitative imaging biomarker. By providing a holistic assessment of the spatial heterogeneity of intra-renal fibrosis, it could assist in evaluating disease severity, prognostic stratification, and treatment monitoring, offering clinicians comprehensive information that surpasses visual assessment and single-parameter metrics. Clinical parameters offer direct pathological relevance (although potentially subjective). Their integration enables a more comprehensive disease characterization. These findings demonstrate machine learning’s exceptional capacity for high-throughput data processing in renal imaging (35, 36). To the best of our knowledge, this is the first study to employ CT radiomics with ML for the noninvasive Oxford T-score evaluation of IgAN.

Statistical analysis of patient demographics and clinical characteristics identified eight statistically significant clinical features (p<0.05) for discriminating Oxford Classification T scores in IgAN: eGFR, Urea, Scr, UA, SBP, DBP, Hb, and 24hTP. Among these, 24-hour proteinuria and eGFR were incorporated into the integrated clinical radiomics model for T-score differentiation. These findings align with previous research (37) and clinical observations. Regular renal function monitoring is essential in patients with primary IgAN. A declining eGFR should prompt clinical vigilance for concurrent irreversible structural damage, particularly in renal interstitial fibrosis. While the correlation between proteinuria and renal interstitial fibrosis is clinically well established, the underlying mechanisms remain incompletely understood. Current evidence suggests that proteinuria may activate tubular cells, inducing the excessive secretion of chemokines and inflammatory mediators that trigger proinflammatory and profibrotic cascades, ultimately leading to renal inflammation and fibrosis (38).

Several limitations of the present study should be acknowledged. First, its retrospective design is inherently susceptible to selection bias. Second, The model was developed using single-center retrospective data, its generalizability to broader populations and different scanning environments still requires confirmation and evaluation through multicenter, prospective external validation datasets. Our findings are chiefly applicable to this specific population, and performance must therefore be re-evaluated in the more commonly encountered cohort of already-treated individuals. Moreover, because the current clinico-radiomics model was constructed predominantly from participants younger than 45 years, extension to a broader age range is imperative to confirm external validity. These issues will constitute the focus of our future research.

In summary, this study developed a diagnostic model integrating pretreatment abdominal CT radiomic features and clinical characteristics with machine learning algorithms to determine Oxford Classification T-scores in patients with IgAN. The combined clinical-radiomics model demonstrated superior diagnostic performance compared with standalone clinical or radiomics models, achieving excellent AUC values (range: 0.94–0.96) across all T-score categories in the testing cohort. These results indicate high accuracy in discriminating the severity of renal interstitial fibrosis in IgAN. By providing clinicians with comprehensive and personalized imaging biomarkers, this model enables the noninvasive assessment of fibrotic progression, offering significant clinical utility for therapeutic decision-making.

## Data Availability

The raw data supporting the conclusions of this article will be made available by the authors, without undue reservation.

## References

[1] El Karoui K Fervenza FC De Vriese AS . Treatment of IgA nephropathy: A rapidly evolving field. J Am Soc Nephrol. (2024) 35:103–16. doi: 10.1681/asn.0000000000000242, PMID: 37772889 PMC10786616

[2] Rajasekaran A Julian BA Rizk DV . IgA nephropathy: an interesting autoimmune kidney disease. Am J Med Sci. (2021) 361:176–94. doi: 10.1016/j.amjms.2020.10.003, PMID: 33309134 PMC8577278

[3] Cattran DC Coppo R Cook HT Feehally J Roberts IS Troyanov S . The Oxford classification of IgA nephropathy: rationale, clinicopathological correlations, and classification. Kidney Int. (2009) 76:534–45. doi: 10.1038/ki.2009.243, PMID: 19571791

[4] Rui Y Yang Z Zhai Z Zhao C Tang L . The predictive value of Oxford MEST-C classification to immunosuppressive therapy of IgA nephropathy. Int Urol Nephrol. (2022) 54:959–67. doi: 10.1007/s11255-021-02974-9, PMID: 34383207

[5] Yamashita N Kramann R . Mechanisms of kidney fibrosis and routes towards therapy. Trends Endocrinol Metab. (2024) 35:31–48. doi: 10.1016/j.tem.2023.09.001, PMID: 37775469

[6] Abbasi M Srivastava A . Advancing noninvasive imaging to quantify kidney fibrosis: hidden in plain sight. Am J Kidney Dis. (2025) 86:560–2. doi: 10.1053/j.ajkd.2025.06.006, PMID: 40639741

[7] Trimarchi H Barratt J Cattran DC Cook HT Coppo R Haas M . Oxford Classification of IgA nephropathy 2016: an update from the IgA Nephropathy Classification Working Group. Kidney Int. (2017) 91:1014–21. doi: 10.1016/j.kint.2017.02.003, PMID: 28341274

[8] Chen T Li X Li Y Xia E Qin Y Liang S . Prediction and risk stratification of kidney outcomes in IgA nephropathy. Am J Kidney Dis. (2019) 74:300–9. doi: 10.1053/j.ajkd.2019.02.016, PMID: 31031086

[9] Amodu A Porteny T Schmidt IM Ladin K Waikar SS . Nephrologists’ Attitudes toward native kidney biopsy: A qualitative study. Kidney Med. (2021) 3:1022–31. doi: 10.1016/j.xkme.2021.06.014, PMID: 34939011 PMC8664729

[10] Milanez T Srinivasan V Premru V Arnol M Ocvirk J Jaimes EA . The safety of percutaneous renal biopsy for acute kidney injury in metastatic renal cell cancer patients with reduced nephron mass. Front Nephrol. (2025) 5:1615779. doi: 10.3389/fneph.2025.1615779, PMID: 40842646 PMC12364680

[11] van Timmeren JE Cester D Tanadini-Lang S Alkadhi H Baessler B . Radiomics in medical imaging-”how-to” guide and critical reflection. Insights Imaging. (2020) 11:91. doi: 10.1186/s13244-020-00887-2, PMID: 32785796 PMC7423816

[12] Hu X Xiao W Wang D Yao J Liu X Xian H . Presence of crescents in IgA nephropathy-prediction from ultrasound images using deep learning. BMC Med Imaging. (2025) 25:411. doi: 10.1186/s12880-025-01958-w, PMID: 41087951 PMC12522557

[13] Meng X Shu J Xia Y Yang R . A CT-based radiomics approach for the differential diagnosis of sarcomatoid and clear cell renal cell carcinoma. BioMed Res Int. (2020) 2020:7103647. doi: 10.1155/2020/7103647, PMID: 32775436 PMC7397414

[14] Uhlig J Leha A Delonge LM Haack AM Shuch B Kim HS . Radiomic features and machine learning for the discrimination of renal tumor histological subtypes: A pragmatic study using clinical-routine computed tomography. Cancers (Basel). (2020) 12 (10):3010. doi: 10.3390/cancers12103010, PMID: 33081400 PMC7603020

[15] Yu B Huang C Fan X Li F Zhang J Song Z . Application of MR imaging features in differentiation of renal changes in patients with stage III type 2 diabetic nephropathy and normal subjects. Front Endocrinol (Lausanne). (2022) 13:846407. doi: 10.3389/fendo.2022.846407, PMID: 35600605 PMC9114464

[16] Chen Z Ying MTC Wang Y Chen J Wu C Han X . Ultrasound-based radiomics analysis in the assessment of renal fibrosis in patients with chronic kidney disease. Abdom Radiol (NY). (2023) 48:2649–57. doi: 10.1007/s00261-023-03965-3, PMID: 37256330

[17] Qin X Xia L Hu X Xiao W Huaming X Xisheng X . A novel clinical-radiomic nomogram for the crescent status in IgA nephropathy. Front Endocrinol (Lausanne). (2023) 14:1093452. doi: 10.3389/fendo.2023.1093452, PMID: 36742388 PMC9895811

[18] Qin X Xia L Zhu C Hu X Xiao W Xie X . Noninvasive evaluation of lupus nephritis activity using a radiomics machine learning model based on ultrasound. J Inflammation Res. (2023) 16:433–41. doi: 10.2147/jir.S398399, PMID: 36761904 PMC9904229

[19] Ren Y Yang F Li W Zhang Y Kang S Cui F . End-to-end CT radiomics-based pipeline for predicting renal interstitial fibrosis grade in CKD patients. Acad Radiol. (2025) 32:3464–74. doi: 10.1016/j.acra.2024.12.050, PMID: 39824728

[20] Gao Y Wang X Zhao X Zhu C Li C Li J . Multiphase CT radiomics nomogram for preoperatively predicting the WHO/ISUP nuclear grade of small (< 4 cm) clear cell renal cell carcinoma. BMC Cancer. (2023) 23:953. doi: 10.1186/s12885-023-11454-5, PMID: 37814228 PMC10561466

[21] Zhan Y Zheng J Chen X Chen Y Fang C Lai C . Developing a CT radiomics-based model for assessing split renal function using machine learning. Jpn J Radiol. (2025) 43:1520–30. doi: 10.1007/s11604-025-01786-6, PMID: 40459698 PMC12397145

[22] Zwanenburg A Vallières M Abdalah MA Aerts H Andrearczyk V Apte A . The image biomarker standardization initiative: standardized quantitative radiomics for high-throughput image-based phenotyping. Radiology. (2020) 295:328–38. doi: 10.1148/radiol.2020191145, PMID: 32154773 PMC7193906

[23] Lambin P Leijenaar RTH Deist TM Peerlings J de Jong EEC van Timmeren J . Radiomics: the bridge between medical imaging and personalized medicine. Nat Rev Clin Oncol. (2017) 14:749–62. doi: 10.1038/nrclinonc.2017.141, PMID: 28975929

[24] van Griethuysen JJM Fedorov A Parmar C Hosny A Aucoin N Narayan V . Computational radiomics system to decode the radiographic phenotype. Cancer Res. (2017) 77:e104–7. doi: 10.1158/0008-5472.Can-17-0339, PMID: 29092951 PMC5672828

[25] Ge XY Lan ZK Lan QQ Lin HS Wang GD Chen J . Diagnostic accuracy of ultrasound-based multimodal radiomics modeling for fibrosis detection in chronic kidney disease. Eur Radiol. (2023) 33:2386–98. doi: 10.1007/s00330-022-09268-3, PMID: 36454259 PMC10017610

[26] Hua C Qiu L Zhou L Zhuang Y Cai T Xu B . Value of multiparametric magnetic resonance imaging for evaluating chronic kidney disease and renal fibrosis. Eur Radiol. (2023) 33:5211–21. doi: 10.1007/s00330-023-09674-1, PMID: 37148348

[27] Bandara MS Gurunayaka B Lakraj G Pallewatte A Siribaddana S Wansapura J . Ultrasound based radiomics features of chronic kidney disease. Acad Radiol. (2022) 29:229–35. doi: 10.1016/j.acra.2021.01.006, PMID: 33589307

[28] Zhu M Ma L Yang W Tang L Li H Zheng M . Elastography ultrasound with machine learning improves the diagnostic performance of traditional ultrasound in predicting kidney fibrosis. J Formos Med Assoc. (2022) 121:1062–72. doi: 10.1016/j.jfma.2021.08.011, PMID: 34452784

[29] Berchtold L Friedli I Vallée JP Moll S Martin PY de Seigneux S . Diagnosis and assessment of renal fibrosis: the state of the art. Swiss Med Wkly. (2017) 147:w14442. doi: 10.4414/smw.2017.14442, PMID: 28634969

[30] Ferguson T Ravani P Sood MM Clarke A Komenda P Rigatto C . Development and external validation of a machine learning model for progression of CKD. Kidney Int Rep. (2022) 7:1772–81. doi: 10.1016/j.ekir.2022.05.004, PMID: 35967110 PMC9366291

[31] Su CT Chang YP Ku YT Lin CM . Machine learning models for the prediction of renal failure in chronic kidney disease: A retrospective cohort study. Diagnost (Basel). (2022) 12 (10):2454. doi: 10.3390/diagnostics12102454, PMID: 36292142 PMC9600783

[32] Belur Nagaraj S Pena MJ Ju W Heerspink HL . Machine-learning-based early prediction of end-stage renal disease in patients with diabetic kidney disease using clinical trials data. Diabetes Obes Metab. (2020) 22:2479–86. doi: 10.1111/dom.14178, PMID: 32844582 PMC7756814

[33] Kanda E Epureanu BI Adachi T Kashihara N . Machine-learning-based Web system for the prediction of chronic kidney disease progression and mortality. PloS Digit Health. (2023) 2:e0000188. doi: 10.1371/journal.pdig.0000188, PMID: 36812636 PMC9931312

[34] Bai Q Su C Tang W Li Y . Machine learning to predict end stage kidney disease in chronic kidney disease. Sci Rep. (2022) 12:8377. doi: 10.1038/s41598-022-12316-z, PMID: 35589908 PMC9120106

[35] Han X Zheng X Wang Y Sun X Xiao Y Tang Y . Random forest can accurately predict the development of end-stage renal disease in immunoglobulin a nephropathy patients. Ann Transl Med. (2019) 7:234. doi: 10.21037/atm.2018.12.11, PMID: 31317004 PMC6603361

[36] Schena FP Anelli VW Trotta J Di Noia T Manno C Tripepi G . Development and testing of an artificial intelligence tool for predicting end-stage kidney disease in patients with immunoglobulin A nephropathy. Kidney Int. (2021) 99:1179–88. doi: 10.1016/j.kint.2020.07.046, PMID: 32889014

[37] Roberts IS Cook HT Troyanov S Alpers CE Amore A Barratt J . The Oxford classification of IgA nephropathy: pathology definitions, correlations, and reproducibility. Kidney Int. (2009) 76:546–56. doi: 10.1038/ki.2009.168, PMID: 19571790

[38] Cheung CK Alexander S Reich HN Selvaskandan H Zhang H Barratt J . The pathogenesis of IgA nephropathy and implications for treatment. Nat Rev Nephrol. (2025) 21:9–23. doi: 10.1038/s41581-024-00885-3, PMID: 39232245 PMC7616674

